# Impact of Reperfusion on Plasma Oxylipins in ST-Segment Elevation Myocardial Infarction

**DOI:** 10.3390/metabo14010019

**Published:** 2023-12-27

**Authors:** Zahra Solati, Arun Surendran, Harold M. Aukema, Amir Ravandi

**Affiliations:** 1 Precision Cardiovascular Medicine Group, St. Boniface Hospital, Albrechtsen Research Centre, Winnipeg, MB R2H 2A6, Canadaharold.aukema@umanitoba.ca (H.M.A.); 2 Department of Physiology and Pathophysiology, Rady Faculty of Health Sciences, University of Manitoba, Winnipeg, MB R3E 0T5, Canada; 3 Department of Food and Human Nutritional Sciences, University of Manitoba, Winnipeg, MB R3T 2N2, Canada; 4 Canadian Centre for Agri-Food Research in Health and Medicine, St. Boniface Hospital Research Centre, Winnipeg, MB R2H 2A6, Canada; 5 Section of Cardiology, University of Manitoba, Winnipeg, MB R3T 5V6, Canada

**Keywords:** reperfusion, lipidomics, oxylipins, inflammation

## Abstract

ST-segment elevation myocardial infarction (STEMI) occurs as a result of acute occlusion of the coronary artery. Despite successful reperfusion using primary percutaneous coronary intervention (PPCI), a large percentage of myocardial cells die after reperfusion, which is recognized as ischemia/reperfusion injury (I/R). There are rapid changes in plasma lipidome during myocardial reperfusion injury. However, the impact of coronary artery reperfusion on plasma oxylipins is unknown. This study aimed to investigate alterations in the oxylipin profiles of STEMI patients during ischemia and at various reperfusion time points following PPCI. Blood samples were collected from patients presenting with STEMI prior to PPCI (Isch, n = 45) and subsequently 2 h following successful reperfusion by PPCI (R-2 h, n = 42), after 24 h (R-24 h, n = 44), after 48 h (R-48 h, n = 43), and then 30 days post PPCI (R-30 d, n = 29). As controls, blood samples were collected from age- and sex-matched patients with non-obstructive coronary artery disease after diagnostic coronary angiography. High-performance liquid chromatography–mass spectrometry (HPLC-MS/MS) using deuterated standards was used to identify and quantify oxylipins. In patients presenting with STEMI prior to reperfusion (Isch group), the levels of docosahexaenoic acid (DHA)-derived oxylipins were significantly higher when compared with controls. Their levels were also significantly correlated with the peak levels of creatine kinase (CK) and troponin T(TnT) before reperfusion (CK: r = 0.33, *p* = 0.046, TnT: r = 0.50, *p* = 1.00 × 10^−3^). The total concentrations of oxylipins directly produced by 5-lipoxygenase (5-LOX) were also significantly elevated in the Isch group compared with controls. The ratio of epoxides (generated through epoxygenase) to diols (generated by soluble epoxide hydrolysis (sEH)) was significantly lower in the Isch group compared with the controls. Following reperfusion, there was an overall reduction in plasma oxylipins in STEMI patients starting at 24 h post PPCI until 30 days. Univariate receiver operating characteristic (ROC) curve analysis also showed that an elevated ratio of epoxides to diols during ischemia is a predictor of smaller infarct size in patients with STEMI. This study revealed a large alteration in plasma oxylipins in patients presenting with STEMI when compared with controls. Total oxylipin levels rapidly reduced post reperfusion with stable levels reached 24 h post reperfusion and maintained for up to 30 days post infarct. Given the shifts in plasma oxylipins following coronary artery reperfusion, further research is needed to delineate their clinical impact in STEMI patients.

## 1. Introduction

Acute coronary syndrome (ACS) is a term used to describe a group of myocardial ischemic conditions, including unstable angina, non-ST elevation myocardial infarction (NSTEMI), and ST-elevation myocardial infarction (STEMI) [1]. While these conditions may have similar clinical symptoms, they differ based on the presence or absence of ST-segment elevation on an electrocardiogram (ECG) and the levels of cardiac markers [2]. Unstable angina refers to an ischemic condition without elevated cardiac enzymes or ST-segment elevation. NSTEMI, on the other hand, is characterized by elevated cardiac biomarkers without ST-segment elevation. STEMI, the most severe form, is identified by elevated ST-segments on an ECG and increased concentrations of cardiac markers, such as troponin T levels [3].

Acute myocardial infarction (MI), encompassing both STEMI and NSTEMI, is one of the leading causes of morbidity and mortality worldwide [4]. For instance, STEMI affects a population of over 3 million individuals globally per year [5]. According to the American Heart Association, there are approximately 605,000 new cases of myocardial infarction annually in the United States, along with 200,000 recurrent cases, translating to approximately 1 myocardial infarction every 40 s [6]. The primary cause of STEMI is either the rupture or erosion of atherosclerotic plaque, which leads to the blockage of the epicardial coronary artery, resulting in transmural ischemia [7]. Timely reperfusion, using primary percutaneous coronary intervention (PPCI), is considered as a standard therapy to reduce clinical outcomes in patients presenting with STEMI [8]. However, reversible and irreversible damage can occur during reperfusion, which is referred to as ischemia/reperfusion (I/R) injury. I/R injury is thought to be responsible for 50% of the final infarct size [9] through several mechanisms: lack of oxygen during ischemia results in nutrient deprivation, metabolic acidosis, hyperkalemia, and calcium overload [10]. Abrupt oxygen introduction following reperfusion leads to the formation of reactive oxygen species (ROS), as a consequence of antioxidant defence impairment. This results in endothelial dysfunction, DNA damage, the activation of inflammatory responses, and eventually cell death [10].

Oxylipins are a group of oxidized lipids that are produced mainly following the activation of phospholipase A2 (PLA2) in response to inflammatory conditions [11]. Polyunsaturated fatty acids (PUFAs), which are chains of hydrocarbons with two or more double bonds [7], are released from the sn-2 positions of membrane phospholipids by phospholipase A2 [11].

Released fatty acids can be further oxidized through three main enzymatic pathways including cyclooxygenase (COX), lipoxygenase (LOX), and cytochrome P450 (CYP450) [12]. Oxylipins exhibit various biological properties depending on the generation pathway. Cyclooxygenase (COX) enzymes, including COX-1 and COX-2, convert polyunsaturated fatty acids into prostanoids, such as prostaglandins (PGs) and thromboxanes (TXs) [13]. These prostanoids interact with the cell surface and intracellular receptors, such as G protein-coupled receptors and Peroxisome Proliferator-Activated Receptor Gamma (PPARγ) to mediate their effects [14]. Lipoxygenase (LOX) enzymes, particularly 5/12/15-LOX, oxidize free arachidonic acid (AA) to produce 4-series leukotrienes and hydroxyeicosatetraenoic acids (HETE) [15]. Eicosapentaenoic acid (EPA) and docosahexaenoic acid (DHA) can also be metabolized by lipoxygenase enzymes to generate specific oxylipins, such as 5-series leukotrienes and hydroxyeicosapentaenoic acids (HEPE) [16]. Lipoxygenase (LOX)-derived oxylipins also mediate their effects by interacting with G protein-coupled receptors and intracellular effectors [16]. Cytochrome P450 (CYP450) enzymes, known for their role in xenobiotic metabolism, are a diverse group of membrane-bound enzymes present within all cells and have epoxygenase or ω-hydroxylase activities [17]. The epoxygenases within the cytochrome P450 pathway (CYP-e) oxidize arachidonic acids (AA) to epoxyeicosatrienoic acids (EpETrE), which can be further converted to dihydroxyeicosatrienoic acids (DiHETrE) by the soluble epoxide hydrolase (sEH) enzyme [18]. Additionally, the cytochrome P450 (CYP450) epoxygenases oxidize linoleic acids (LA) to epoxyoctadecenoic acids (EpOMEs), as well as eicosapentaenoic acid (EPA) and docosahexaenoic acid (DHA) to epoxyeicosatetraenoic acids (EpETE) and epoxydocosapentaenoic acids (EpDPE), respectively [19]. These oxylipins can further undergo hydrolysis or conversion to diols by the soluble epoxide hydrolase (sEH) enzyme. Cytochrome P450 hydroxylases (CYP-h) are other groups of enzymes in this pathway that produce HETEs from arachidonic acid (AA), HEPEs, and HDoHEs from eicosapentaenoic acid (EPA) and docosahexaenoic acid (DHA), respectively [20,21]. Oxylipins derived from the cytochrome P450 (CYP450) pathway can interact with various receptors, including but not limited to G protein-coupled receptors (GPCRs) and nuclear receptors [18].

The biological effects of oxylipins depend not only on the oxidative pathway but also on the fatty acid precursors [11]. Oxylipins derived from n-6 poly-unsaturated fatty acids (PUFAs), such as arachidonic acid (AA) and linoleic acid (LA), can initiate inflammation, change ion channel functions, alter transcriptional programming, and cause ventricular remodelling, which can all contribute to I/R injury [22,23]. However, evidence from experimental and clinical studies indicates that oxylipins derived from n-3 poly-unsaturated fatty acids (PUFA) have anti-inflammatory, anti-arrhythmic and cardio-protective properties [24]. Previous studies have examined the roles of limited oxylipins in I/R injury in animal models [25]. However, a comprehensive analysis of the oxylipin profile in STEMI patients has not been well studied. Hence, this study aimed to investigate alterations in oxylipin profiles of STEMI patients during ischemia and various time points of reperfusion following PPCI.

## 2. Materials and Methods

### 2.1. Study Population

**Figure 1:**
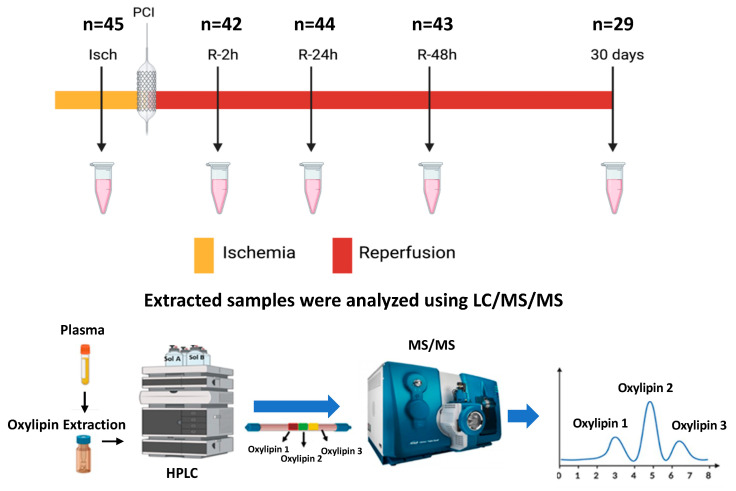
Overall study design. Plasma samples were collected at five different time points: pre-PPCI (Isch), 2 h post PPCI (R-2 h), 24 h post angioplasty (R-24 h), 48 h post angioplasty (R-48 h), and 30 days following PPCI (R-30 d).

Study participants: Patients were from the St. Boniface Hospital Cardiac Cath laboratories and presented with STEMI. All patients were aged above 18 years old, had STEMI on 12 lead ECG, presented with chest pain, and had an occluded coronary artery at baseline as diagnosed by coronary angiography. Age- and sex-matched controls were recruited from patients who were referred for coronary angiography but did not have any evidence of coronary disease. Patients with a history of congestive heart failure, diabetes, end-stage renal disease in dialysis, autoimmune disease, and valvular heart disease were excluded. Blood samples were collected from 45 patients presenting with STEMI by venipuncture at various time points: at presentation before PPCI (Ischemic phase) (Isch, n = 45), 2 h following successful reperfusion by PPCI (R-2 h, n = 42), after 24 h (R-24 h, n = 44), after 48 h (R-48 h, n = 43), and then 30 days post PPCI (R-30 d, n = 29). The selection of time points for blood collection after PPCI was determined based on the hospital schedule for the patient’s blood draw. It is important to note that the number of samples collected and analyzed in each group may vary due to sample loss. All samples were collected in EDTA venipuncture tubes, centrifuged at 3000 rpm for 10 min to isolate plasma, and were kept at −80 °C freezer until analysis. Blood samples from control patients were collected following angiography (n = 44). The study was approved by the University of Manitoba and the St. Boniface Hospital Research Ethics Board. The overall study design is shown in Figure 1.

### 2.2. Free Oxylipin Analysis

An amount of 150 µL of plasma was used for oxylipin analysis. After solid-phase extraction, 40 uL of the extracted sample was separated on Luna 5u C18(2) 100 A; 250 × 2.00 mm (Phenomenex). Solvent A comprised water–acetonitrile–acetic acid (70:30:0.02) and solvent B comprised acetonitrile–isopropanol (50:50) at a flow rate of 325 uL/min with the column oven at 50 Celcius. The program consisted of 100% A at time 0, 3 min 25% solvent B, 11 min 45% Solvent B, 13 min 60% B, 18 min 75% B, 18.5 min 90% B, 21 min 0% B. The HPLC was in line with MS/MS (AB SCIEX 4000 QTRAP Framingham, MA, USA) with multiple-reaction monitoring (MRM) as previously described [26,27]. Details of the deuterated internal standards, collision-induced dissociation mass transitions, retention times, detector response factors, and the lower/upper limit of detection for analytes can be found in the Supplementary Material (Table S1). The quantification limit was set at 5 times above the background. Quantification of oxylipins was determined using the stable isotope dilution method [28] and expressed as nanomolar (nM). The raw MS/MS data are available in in the Supplementary Material.

### 2.3. Statistical Analysis

Data were analyzed using Origin (version 17, USA). A multivariate pattern recognition tool, namely partial least squares–discriminant analysis (PLS-DA), was applied to understand the changes in plasma oxylipin levels during I/R using the metaboanalyst website (https://www.metaboanalyst.ca/) (accessed 1 December 2023). Oxylipin values were treated as continuous variables and assessed for Gaussian distribution using Shapiro–Wilk’s test. Student’s *t*-test was used to compare the means between two groups. One-way analysis of variance (ANOVA) with a post hoc Tukey test for multiple comparisons was used to determine statistical significance between study groups. Associations between specific oxylipins and creatine kinase (CK) and high-sensitivity troponin T (TnT) levels were assessed by applying Pearson correlations. Receiver operating characteristic (ROC) curve analysis (using the metaboanalyst website (https://www.metaboanalyst.ca/) (accessed 1 December 2023)) was used to evaluate the potential diagnostic capability of oxylipins. All data are presented as mean ± SEM. *p*-value < 0.05 was considered to be statistically significant.

## 3. Results

### 3.1. Patient Characteristics

The characteristics of controls and STEMI patients along with laboratory data and cardiac injury markers, including creatine kinase (CK) and troponin T (TnT), are presented in Table 1. In this study, 66% of the STEMI population and 56% of the controls were male (*p* = 0.08). The mean age was 65 ± 2 years in the STEMI population and 60 ± 1 year in control subjects (*p* = 0.06). The average body mass index (BMI) was significantly different between STEMI and control populations (25.80 ± 1.14 and 30 ± 1, respectively, *p* = 0.001). Based on the laboratory data, the STEMI patients had normal triglycerides (TG), cholesterol (TC), and low- and high-density lipoprotein (LDL and HDL). There were no significant differences regarding the use of angiotensin-converting enzyme inhibitors/angiotensin-receptor blockers (ACEIs/ARBs), beta-blockers, and statins between the STEMI and control groups (Table 1). The median ischemic time (from the onset of chest pain to reperfusion) was 150 min. A total of 50% of participants had a right coronary artery (RCA) infarct, whereas 42% and 8% had left anterior descending coronary artery (LAD) and circumflex coronary artery occlusions, respectively. The prevalence of type 2 diabetes (21%), hypertension (42%), and dyslipidemia (42%) at presentation to the hospital were not significantly different in STEMI patients compared with controls (Table 1).

### 3.2. Overall Characterization of Plasma Oxylipins Levels in STEMI Patients and Controls

**Figure 2:**
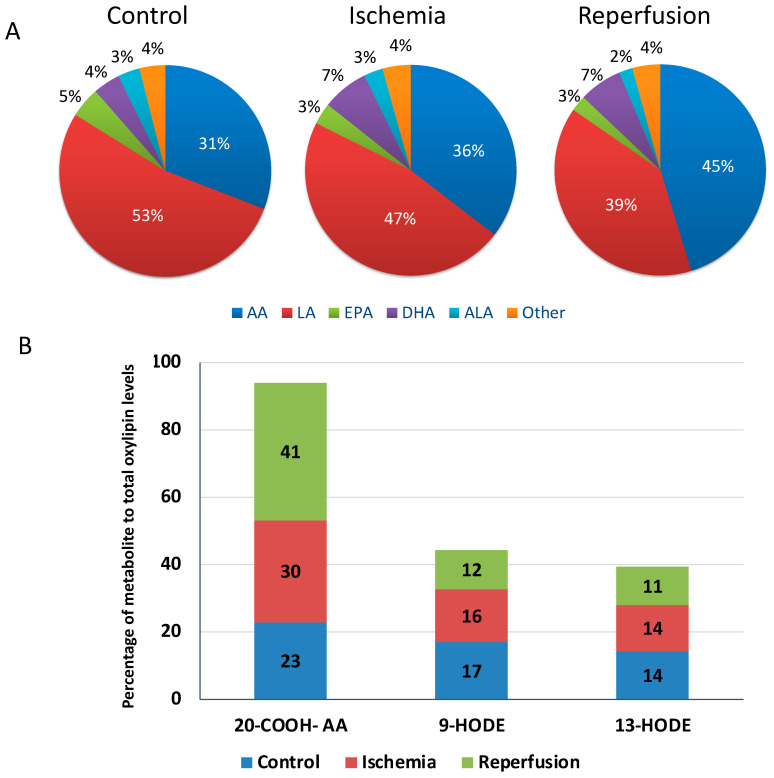
Distribution of plasma oxylipins. (**A**) Percent distribution of oxylipins based on fatty acid precursors in control and STEMI patients during ischemia and in reperfusion. (**B**) Change in distribution of 20-COOH-AA, 9-HODE and 13-HODE oxylipins in control patients compared to STEMI patients during ischemia and reperfusion. Reperfusion values represent the average of all reperfusion time points. Abbreviations—AA: arachidonic acid; ALA: alpha-linoleic acid; DHA: docosahexaenoic acid; EPA: eicosapentaenoic acid; HODE: hydroxy-octadecadienoic acid; LA: linoleic acid; 20-COOH-AA: 20-carboxy-arachidonic acid.

**Figure 3:**
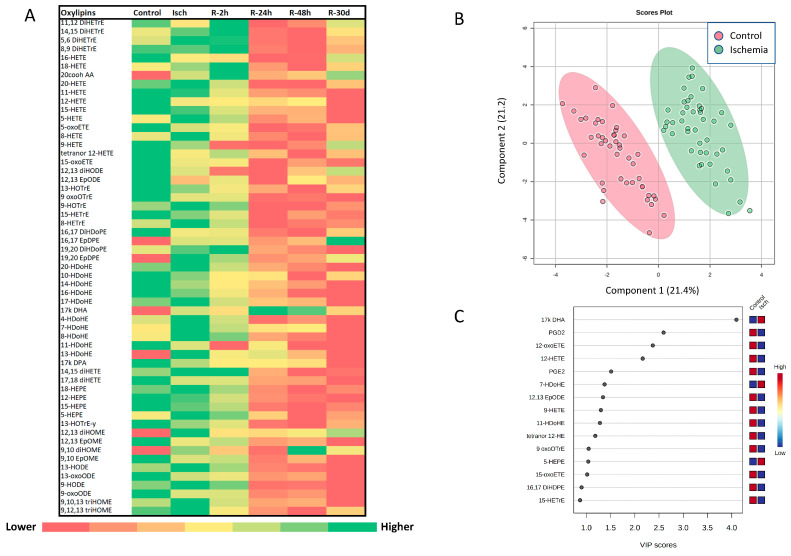
The pattern of oxylipin alterations during I/R compared with control patients. (**A**) Heat map of plasma oxylipin concentrations comparing controls and STEMI patients at 1 time point before reperfusion and 4 time points post reperfusion up to 30 days. (**B**) PLS-DA scores plots for comparison of the oxylipin profiles in the Isch and control groups. (**C**) VIP score for compounds that had the most contributions in PLS-DA separation between Isch and control group. Metaboanalys website was used for PLS-DA and VIP score analyses. Abbreviations—PLS-DA: partial least squares–discriminant analysis; VIP: variable’s importance.

Sixty oxylipins were present at quantifiable levels in the plasma of controls: 53% and 31% of these were derived from linoleic acid (LA) and arachidonic acid (AA), respectively (Figure 2A). Fifty-seven oxylipin metabolites were quantified in the plasma of STEMI patients. Linoleic acid (LA)-derived oxylipins constituted 47% and 39% of total quantifiable oxylipins during ischemia and reperfusion (average of reperfusion time points), respectively (Figure 2A). Arachidonic acid (AA)-derived oxylipins accounted for 36% and 45% of total quantifiable oxylipins during the ischemic episode and reperfusion, respectively (Figure 2A). The percentage of oxylipins derived from other fatty acid precursors are presented in Figure 2A. Heatmap analysis of all quantified oxylipins in our participants revealed a trend for higher oxylipin levels in controls during ischemia (Figure 3A). The 20-carboxy-arachidonic acid (20-COOH-AA), an arachidonic acid-derived oxylipin, along with 9- and 13-hydroxyoctadecadienoic acid (HODE), linoleic acid-derived metabolites, were the most abundant oxylipins in the plasma of our participants (Figure 2B).

### 3.3. Alterations in the Plasma Oxylipin Profile of Ischemic STEMI Patients (Isch Group) Compared to Controls

**Figure 4:**
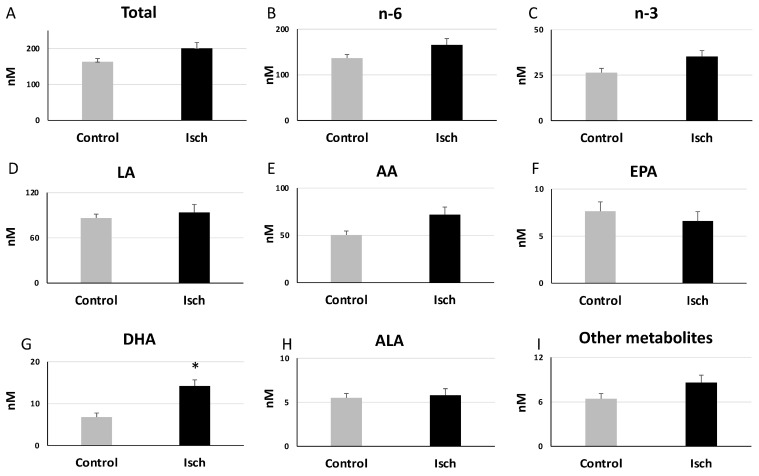
Changes in levels of oxylipin groups ((**A**–**I**), based on FA precursors) in Isch and control groups. Values are means ± SEM. * Statistically significant difference compared with the control group (Student’s *t*-test, *p* < 0.05). Abbreviations—AA: arachidonic acid; ALA: alpha-linoleic acid; DHA: docosahexaenoic acid; EPA: eicosapentaenoic acid; LA: linoleic acid.

When comparing the total oxylipin levels between the control and Isch groups, there was a trend for higher oxylipin concentrations in the Isch group compared with the controls (Isch: 200.87 ± 16.00 nM, Control:163.19 ± 8.90 nM) (Figure 4A), but this difference did not reach statistical significance. Categorizing oxylipins based on their fatty acid precursors showed that among all oxylipin groups, only the total levels of docosahexaenoic acid (DHA)-derived oxylipins were significantly elevated in the Isch group compared with the control group (Isch: 14.23 ± 1.46, Control: 6.79 ± 0.96 nM, *p* = 9.18 × 10^−5^) (Figure 4G). Their levels were also significantly correlated with peak levels of creatine kinase (CK) and troponin T (TnT) (total of DHA-derived oxylipins: CK: r = 0.33, *p* = 0.046, TnT: r = 0.50, *p* = 1.00× 10^−3^). PLS-DA analysis also showed a clear distinction in the oxylipin profile between the Isch and control groups (Figure 3B). Among all oxylipins, a docosahexaenoic acid metabolite, namely 17-keto DHA, contributed the most to this differentiation, as indicated by its variable importance (VIP) score in the PLS-DA model (Figure 3C). Interestingly, eleven out of fourteen metabolites that had significant correlations with the peak levels of CK and TnT were also DHA-derived metabolites (Table 2).

We also compared the total levels of oxylipins based on their generation pathways. As our controls and STEMI patients were on acetylsalicylic acid (ASA) therapy, only low levels of cyclooxygenase-derived oxylipins were present. Of these, prostaglandin D_2_ (PGD_2_) and PGE_2_ were present only in the control groups (Table 3).

**Figure 5:**
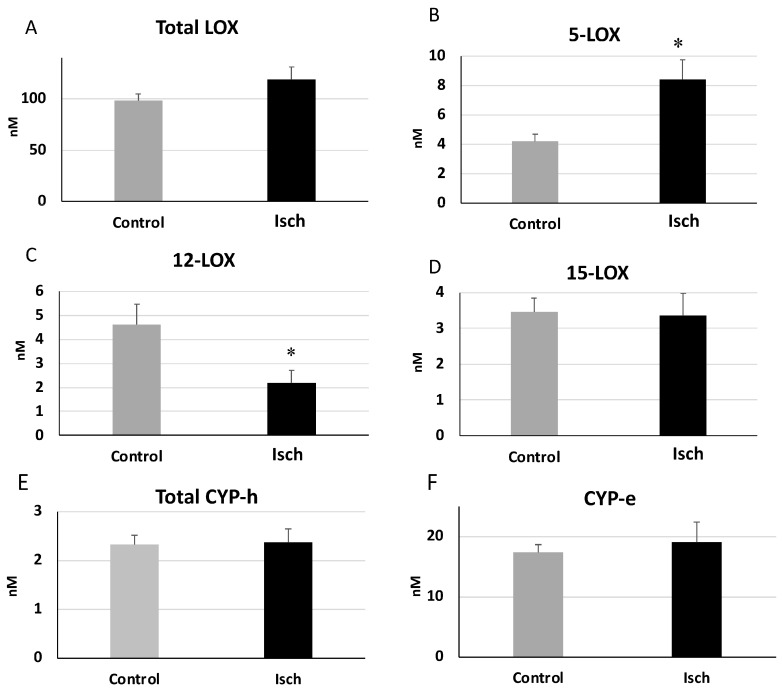
Total levels of oxylipins generated through LOX, CYP-h, and CYP-e (**A**–**F**) pathways in Isch and control patients. Values are means ± SEM. * Statistically significant difference compared with the control group (Student’s *t*-test, *p* < 0.05). Abbreviations—LOX: lipoxygenase; CYP-h: cytochrome P450 hydroxylase.

The total levels of the oxylipins generated through the lipoxygenase (LOX) pathway did not significantly differ between the Isch and control groups (Isch: 118.72 ± 12.19, Control:131.10 ± 8.20 nM) (Figure 5A), but sub-categorizing them based on 5/12/15 LOX pathways revealed that the total levels of the oxylipins generated through the 5-LOX pathway (including 5-HETE, 5-HEPE, 4-HDoHE, 7-HDoHE) were significantly higher in the Isch group compared with controls (Isch = 8.39 ± 1.35, Control: 4.18 ± 0.50 nM, *p* = 0.004946) (Figure 5B). In contrast, oxylipins generated through the 12-LOX pathway (12-HETE, 12-HEPE, 14-HDoHE) were significantly lower in ischemia (Isch group) compared with controls (Isch:2.19 ± 0.52, Control: 4.61 ± 0.86 nM, *p* = 0.017585) (Figure 5C). Oxylipins produced via the 15-LOX pathway (15-HETE, 15-HETrE, 15-HEPE, 17-HDoHE) were not significantly different between the Isch and control groups (Isch: 3.35 ± 0.61, Control: 3.45 ± 0.38) (Figure 5D). Oxylipins generated via the cytochrome P450 hydroxylases (CYP-h) pathway (16-HETE, 17-HETE, 18-HETE, 18-HEPE, and 20-HETE, 20-HDoHE) and the cytochrome P450 epoxygenase (CYP-e) did not significantly differ between the Isch and control groups.

### 3.4. Impact of Reperfusion on Plasma Oxylipin Profile

**Figure 6:**
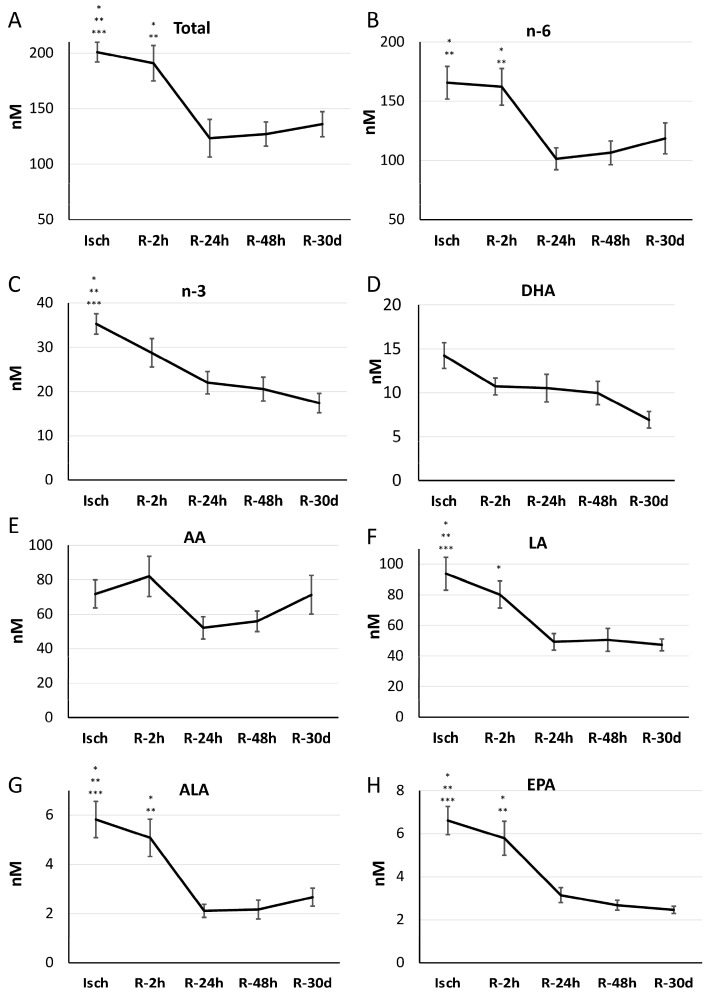
Alteration in levels of total oxylipins and oxylipin groups based on fatty acid precursors (**A**–**H**) in STEMI patients during 30 days post MI. Values are means ± SEM. * Significant difference from R-24 h. ** Significant difference from R-48 h. *** Significant difference from R-30 d (one-way analysis of variance (ANOVA) with a post hoc Tukey test, *p* < 0.05). Abbreviations—AA: arachidonic acid; ALA: alpha-linoleic acid; DHA: docosahexaenoic acid; EPA: eicosapentaenoic acid; LA: linoleic acid.

We were next interested in the impact of myocardial reperfusion on the levels of oxylipins in STEMI patients. Total levels of oxylipins were significantly elevated during the ischemic period (Isch) and early after reperfusion (R-2 h) compared to other reperfusion groups. Subsequently, their concentrations decreased significantly at 24 h after reperfusion and then remained unchanged until 30 days post MI (Isch: 200.87 ± 16.00. R-2 h: 190.87 ± 17.00, R-24 h: 123.34 ± 10.87, R-48 h: 127.01 ± 11.28, and R-30 d: 135.95 ± 13.88 nM, *p* = 1.85 × 10^−5^) (Figure 6A).

Oxylipins were then categorized based on their fatty acid precursors. n-6- and n-3-derived oxylipins were reduced by 24 h of reperfusion compared to ischemic levels (total n-6 metabolites: Isch: 165.59 ± 13.81, R-2 h: 162.12 ± 15.51, R-24 h: 101.34 ± 9.19, R-48 h: 106.49 ± 10.03, and R-30 d: 118.55 ± 13.03 nM, *p* = 2.28× 10^−5^, (Figure 6B)) (total n-3 metabolites: Isch: 35.28 ± 3.20, R-2 h: 28.74 ± 2.53, R-24 h: 22.00 ± 2.69, R-48 h: 20.58 ± 2.18, and R30 d: 17.39 ± 1.60 nM, *p* = 1.69× 10^−9^ (Figure 6C)). Individual fatty acids reflected similar patterns and were significant for linoleic acid (LA), alpha-linoleic acid (ALA), and eicosapentaenoic acid (EPA) oxylipins but not for arachidonic acid (AA) and docosahexaenoic acid (DHA) oxylipins (Figure 6D–H).

**Figure 7:**
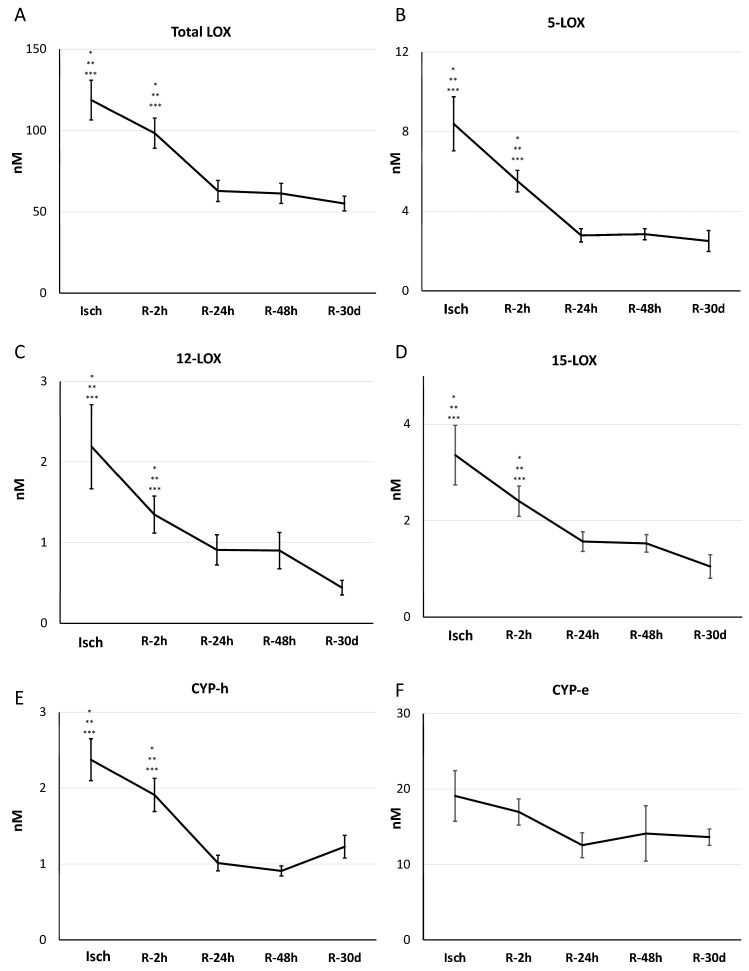
Total levels of oxylipins that were generated through LOX and CYP pathways in STEMI patients during ischemia and 4 time points subsequent to reperfusion. (**A**–**F**) Values are means ± SEM. * Significant difference from R-24 h. ** Significant difference from R-48 h. *** Significant difference from R-30 d (one-way analysis of variance (ANOVA) with a post hoc Tukey test for multiple comparisons, *p* < 0.05). Abbreviations—CYP-e: vytochrome P450 epoxygenase; CYP-h: cytochrome P450 hydroxylase; LOX: lipoxygenase.

Categorizing oxylipins based on their generation pathways also showed similar patterns. Total oxylipins produced via the lipoxygenase (LOX) pathway decreased significantly post reperfusion for 24 h (Isch: 118.72 ± 12.19, R-2 h: 98.40 ± 9.20, R-24 h: 62.84 ± 6.48, R-48 h: 61.33 ± 6.18, and R-30 d: 55.10 ± 4.55 nM, *p* = 1.68 × 10^−7^) (Figure 7A). Sub-categorizing the lipoxygenase (LOX) pathway revealed that total levels of metabolites that were generated through the 5/12/15 LOX pathways also decreased significantly after 24 h of reperfusion (5-LOX: Isch: 8.39 ± 1.35, R-2 h: 5.51 ± 0.54, R-24 h: 2.79 ± 0.33, R-48 h: 2.85 ± 0.27, and R-30 d: 2.51 ± 0.52 nM, *p* = 2.6666 × 10^−8^; Figure 7B) (12-LOX: Isch: 2.19 ± 0.52, R-2 h: 1.34 ± 0.22, R-24 h: 0.91 ± 0.18, R-48 h:0.90 ± 0.22, and R-30 d: 0.44 ± 0.09 nM, *p* = 0.0022826; Figure 7C) (15-LOX: Isch: 3.35 ± 0.61, R-2 h: 2.40 ± 0.31, R-24 h: 1.56 ± 0.20, R-48 h: 1.52 ± 0.18, and R-30 d: 1.04 ± 0.24 nM, *p* = 0.00015374; Figure 7D).

The levels of oxylipins that were produced through cytochrome P450 hydroxylases (CYP-h) decreased significantly following reperfusion (Isch: 2.37 ± 0.27, R-2 h: 1.91 ± 0.21, R-24 h: 1.01 ± 0.10, R-48 h: 0.9 ± 0.06, and R-30 d: 1.22 ± 0.15 nM, *p* = 1.49 × 10^−8^).

**Figure 8:**
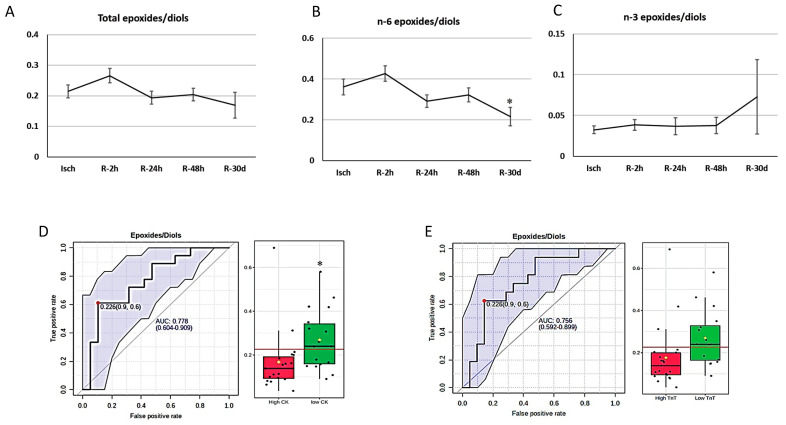
Changes in ratios of epoxides/diols in STEMI patients during ischemia and 4 time points subsequent to reperfusion and correlation with CK and Troponin T. (**A**) Total, (**B**) n-6, and (**C**) n-6 epoxides/diols in ischemia and 30 days post reperfusion (one-way analysis of variance (ANOVA) with a post hoc Tukey test, *p* < 0.05). (**D**) ROC curve analysis based on TnT levels and (**E**) levels of epoxides/diols in patients with “low TnT” and “high TnT” levels. The box shows the 25th and 75th percentiles. The whiskers are the 5th and 95th percentiles. * Significant difference from Isch and R-2 h (*p* < 0.05). We utilized Metaboanalyst (https://www.metaboanalyst.ca/) (accessed 1 December 2023) for biomarker analysis with classical univariate ROC curve analyses. The cutoff = 0.226 was selected as the most optimal cutoff for both CK and TnT. With this cutoff, the sensitivity and specificity are as follows. CK—cutoff: 0.226, sensitivity: 0.611 (confidence interval: 0.383–0.833), specificity: 0.895 (confidence interval: 0.737–1). TnT—cutoff: 0.226, sensitivity: 0.625 (confidence interval: 0.438–0.812), specificity: 0.895 (confidence interval: 0.714–0.952). Abbreviations—CK: creatine kinase; TnT: troponin T.

However, the 20-HETE metabolite, 20-COOH-AA, remained elevated during the 30-day reperfusion period (Table S2). For cytochrome P450 epoxygenase (CYP-e) oxylipins, there were no changes (Figure 7E,F), but the ratio of n-6 (but not n-3)-derived epoxides/diols was reduced significantly 30 days after reperfusion (ratio of n-6-derived epoxides/diols: Isch: 0.36 ± 0.03, R-2 h: 0.42 ± 0.02, R-24 h: 0.29 ± 0.03, R-48 h: 0.32 ± 0.03, and R-30 d: 0.21 ± 0.04, *p* = 0.0042022) (Figure 8A–C). Alterations in the concentrations of all individual metabolites during I/R are presented in Table S2.

### 3.5. Oxylipins and Cardiac Biomarkers

STEMI patients were classified based on the median of creatine kinase (CK) and troponin T (TnT) levels. Patients with above-median levels of creatine kinase (CK) and troponin T (TnT) were considered as “high CK” or “high TnT” groups and patients with below-median levels of CK and TnT were identified as “low CK” and “low TnT” groups (the median of peak CK: 1111.5 ng/L; the median of peak TnT: 2189 ng/L). ROC curve analysis was used to identify potential biomarkers based on the area under the curve (AUC) using the Metaboanalys website. We found that the total-epoxides-to-diols ratio predicts low infarct size, with AUC = 0.77 (*p* = 0.03) based on CK levels and AUC = 0.76 (*p* = 0.054) based on TnT levels (Figure 8D,E).

## 4. Discussion

Oxylipins are a group of oxidized lipids that can be generated following the activation of membrane enzymes, such as phospholipase A2 (PLA2) [29]. Previous studies showed the activation of membrane enzymes, such as phospholipase A2 during I/R [30]. Acute phospholipase A2 activation may lead to the breakdown of membrane phospholipids, the release of polyunsaturated fatty acids, and subsequently the generation of oxylipins [21]. Oxylipins play a significant role in I/R injury, exerting diverse biological effects. For example, oxylipins derived from n-6 fatty acids, such as arachidonic acids and linoleic acids, contribute to inflammation, alter ion channel functions, and impact ventricular remodelling [22]. On the other hand, oxylipins derived from n-3 fatty acids possess anti-inflammatory, anti-arrhythmic, and cardio-protective properties [31]. The main goal of this study was to address the following key inquiries: How does myocardial I/R affect the oxylipin profile in STEMI patients? Do the alterations in the plasma oxylipin concentrations correlate with the levels of cardiac injury markers? Could the magnitude of I/R injury be predicted based on the baseline levels of an oxylipin metabolite or group? To respond to these questions, we analyzed the plasma oxylipin profile of our STEMI samples using HPLC-MS/MS.

The linoleic acid-derived oxylipins were the dominant oxylipin group in the plasma of our participants in both STEMI patients (at baseline) and controls. This observation was consistent with the results of previous studies [32] and mainly attributed to the higher consumption of vegetable oils containing linoleic acid in the Western diet [33,34].

Among all quantified oxylipins, 20-COOH-AA, 9-HODE, and 13-HODE were the three most abundant compounds in the plasma of our participants. This finding is indeed in accordance with the results of previous reports. In a study by Quehenberger et al. [35], it was found that 13-HODE and 9-HODE are the most abundant oxylipins quantified in reference material plasma (SRM1950) using a lipidomics approach. However, it is important to note that 20-COOH-AA was not measured in their samples. Another study conducted by Loomba et al. [36] identified 13-HODE, 20-COOH-AA, and 9-HODE as the dominant oxylipins in the plasma of healthy individuals.

To investigate the impact of myocardial ischemia on the oxylipin profile during myocardial I/R, we conducted a comparison of the plasma levels of various oxylipin groups in STEMI patients and controls. Our findings indicate a significant involvement of oxidized metabolites of docosahexaenoic acid (DHA) during myocardial ischemia. Firstly, among the categorized oxylipin groups based on fatty acid precursors, docosahexaenoic acid (DHA)-derived metabolites were the only group of oxylipins that were elevated during the ischemic phase compared with controls. Secondly, the concentrations of docosahexaenoic acid (DHA)-derived metabolites during ischemia (Isch group) were correlated with markers of cardiac injury, specifically creatine kinase (CK) and troponin T (TnT) (Table 3). Thirdly, the largest fold change observed among all oxylipin metabolites was in the levels of 17-keto DHA during ischemia (115-fold change), which was also associated with the highest VIP score in the PLS-DA analysis. Overall, these findings suggest a prominent role of DHA-derived oxylipins during myocardial ischemia.

Increased concentrations of DHA-derived oxylipins following myocardial infarction (MI) were previously reported. In a prospective observational study, the levels of protectins, the docosahexaenoic acid (DHA)-derived oxylipins generated through the lipoxygenase (LOX) pathway, increased significantly during ischemia (before peak troponin T levels) in STEMI patients (n = 15) compared with patients presenting with stable coronary artery disease (CAD) (n = 10) and healthy controls (n = 10) [24]. The authors suggested that the elevated levels of docosahexaenoic acid (DHA)-derived oxylipins during myocardial ischemia would be part of the defensive mechanism aimed to initiate repair, possibly through the anti-inflammatory properties of these oxylipins [37] through interacting with Peroxisome Proliferator-Activated Receptors (PPARs) [38]. It has been demonstrated that increased activities of PPAR-α and PPAR-γ during ischemia–reperfusion can have a protective effect on the myocardium against I/R injury [39,40]. Notably, in our study, 17-keto DHA, which showed the greatest increase in our data, acts as an agonist for both PPAR-α and PPAR-γ receptors [41].

Clinical studies have confirmed the positive effects of supplementation with n-3 fatty acids following myocardial infarction. In a study by Heydari et al. [42], 358 patients were randomly assigned to receive either 4 g/day of omega-3 along with conventional therapy or conventional therapy alone after MI. After 6 months, the group receiving omega-3 supplementation showed significant reductions in left ventricular end-systolic volume indexed (LVESVI) to body surface area (−5.4%) and non-infarct myocardial fibrosis (−2.1%) compared to the control group. n-3 fatty acid supplementation was also associated with significant decreases in the levels of myeloperoxidase and lipoprotein-associated PLA2 as well as the suppression of tumorigenicity 2 (ST2), a marker of myocardial fibrosis [42]. Thus, it was concluded that n-3 fatty acid supplementation improves left ventricular (LV) remodelling and non-infarct myocardial fibrosis by suppressing inflammation at both the systemic and myocardial levels post acute myocardial infarction [42]. In another randomized controlled trial, the effects of adjunctive therapy with n-3 fatty acids (ω-3 PUFA) were assessed in patients with myocardial infarction who underwent PPCI [43]. Sixty patients were randomly assigned to receive either ω-3 PUFA supplementation (2 g/day) in addition to guideline-adjusted therapy (10 mg/day rosuvastatin) or guideline-adjusted therapy alone, within 3–7 days of hospital admission. Over the first 3 months after acute myocardial infarction, lipid profile and endothelial function were evaluated. Additionally, the changes in these variables were found to be positively correlated with changes in the levels of 16,17 EpDPE and EpETE [43].

Based on the findings, it can be concluded that chronic supplementation of n-3 fatty acids has cardioprotective properties in reducing myocardial I/R injury. Since our data showed an acute increase in the levels of docosahexaenoic acid (DHA)-derived oxylipins during the ischemic episode, it may be worth exploring the potential therapeutic option of administering an acute infusion of docosahexaenoic acid (DHA) (in the form of an emulsion) before PPCI, rather than relying on chronic supplementation after myocardial infarction. This approach has been tested in previous preclinical studies [44].

To find out the predominant oxidative pathway during ischemia, we categorized oxylipins based on their respective enzymatic pathways. Overall total levels of the oxylipins produced through the lipoxygenase (LOX) pathway did not differ significantly between the Isch and control groups. However, we found that 5-LOX-derived oxylipins were significantly elevated during ischemia compared with controls. A previous study has indicated that 5-LOX enzymes are activated in myocytes during myocardial I/R [45]. The oxylipins included in our analysis were 5-HETE, 5-HEPE, 4-HDoHE, and 7-HDoHE, which are direct metabolites of this pathway. All of these compounds, with the exception of 5-HETE, belong to the group of n-3-derived oxylipins. These oxylipins have been found to exhibit lower biological activity and less inflammatory effects when compared to compounds derived from arachidonic acid [11]. Based on these data, it can be suggested that the 5-LOX pathway may have been activated during the ischemic phase of STEMI. However, the oxidation primarily occurred in n-3 fatty acids rather than n-6 fatty acids. This can be attributed to the natural defense mechanism of the body, which aims to protect the myocardium against ischemic injury. Further research is needed to better understand the specific roles of 5-LOX pathways in STEMI and their potential implications for therapeutic interventions.

On the contrary, we observed a reduction in the total levels of 12-LOX-derived oxylipins during ischemia (namely 12-HETE, 12-HEPE, 14-HDoHE). It is important to note that oxidized metabolites that can be generated through other lipoxygenase (LOX) pathways, non-enzymatic pathways, or secondary enzymes were excluded from analysis. Upon examining the changing patterns of these three oxylipins, it is evident that only changes in the levels of 12-HETE were found to be statistically significant. 12-HETE is a major platelet-derived eicosanoid, although its role in platelet function remains unclear [46]. It has been previously reported that acetylsalicylic acid (ASA) therapy intake could decrease the production of 12-HETE in a dose-dependent manner. The observed effect seems to be specific to acetylsalicylic acid (ASA) and not a result of secondary effects caused by cyclooxygenase (COX) inhibition [47,48], which is supported by the fact that treatment with indomethacin, a COX-1 inhibitor, does not have any impact on 12-HETE levels [49]. Altogether, we can suggest that the lower levels of 12-HETE in our participants during ischemia can be related to the higher dose of acetylsalicylic acid (ASA) intake in STEMI patients compared with controls.

We did not observe significant differences in the total levels of metabolites generated through the 15-LOX in our study groups. Consistent with our findings, serum levels of 15-HETE were not significantly different in patients who underwent coronary bypass surgery in comparison with controls, although the ALOX-15 enzyme (but not ALOX-12) and its product, 15-HETE, were higher in biopsies from ischemic hearts of these patients compared with non-ischemic heart tissue [50]. These data may suggest that 15-LOX metabolites may increase locally rather than systematically in STEMI patients. Looking at the changing patterns of individual compounds in this group showed that 20-COOH-AA was elevated during ischemia in STEMI patients (Table 2). 20-COOH-AA is a major metabolite of 20-HETE. Although the biological properties of 20-COOH-AA are not well studied, previous studies reported its vasodilatory effects in the porcine coronary artery [51], and it is a dual activator of proliferator-activated receptors, namely, Peroxisome Proliferator-Activated Receptor (PPAR)-α and PPAR-γ receptors, in COS-7 cells [32].

The total levels of oxylipins that were generated through the cytochrome P450 epoxygenase (CYP-e) pathway did not significantly differ between the Isch and control groups. However, the total epoxides/diols ratio reduced significantly during ischemia, which reflects the elevated activity of the soluble epoxide hydrolysis (sEH) enzyme or less degradation of these metabolites during the ischemic episode. Our data also indicate that the ratio of epoxides to diols during ischemia may serve as a potential marker for predicting the size of an infarct. Specifically, we observed that patients with lower ratios of epoxides to diols at baseline tend to have larger infarct sizes. This finding is supported by an area under the curve (AUC) of 0.77 (*p* = 0.03) based on creatine kinase (CK) levels and an area under the curve (AUC) of 0.76 (*p* = 0.054) based on troponin T (TnT) levels, as shown in Figure 8. Based on these results, inhibiting the soluble epoxide hydrolysis (sEH) enzyme before PPCI could be considered as a potential therapeutic option. By inhibiting the soluble epoxide hydrolysis (sEH) enzyme, it may be possible to modulate the epoxide-to-diol ratio and potentially reduce the size of the infarct. Further research and clinical trials are warranted to explore the efficacy and safety of this therapeutic approach.

To summarise, docosahexaenoic acid (DHA)-derived oxylipins were the only group of oxylipins that increased significantly during ischemia in our participants. Considering their reported anti-inflammatory effects, the increments in their levels during the ischemic phase could be part of a body’s defensive mechanism to limit further tissue damage and protect the myocardium against ischemia [24]. Another important observation of this study was the increases in the levels of 17 Keto DHA and 20-COOH-AA during ischemia. These two compounds are the products of dehydrogenase enzymes with reported vasodilatory and anti-inflammatory effects. Therefore, their elevated levels during ischemia may be protective to the myocardium [32,51]. On the other hand, levels of 5-LOX metabolites, rather than 12/15-LOX, were significantly higher during the ischemic episode, which can highlight the importance of this pathway during the ischemic phase of STEMI. According to our data, the ratio of epoxides to diols during the ischemic phase can potentially be used as a marker to predict the size of an infarct. Our observations indicate that patients with lower ratios of epoxides to diols at baseline (ischemia) tend to have larger infarct sizes, which highlights the potential involvement of the soluble epoxide hydrolysis (sEH) enzyme in STEMI patients. It should be mentioned that our control and STEMI population were well matched, but there were differences in BMI, with the control population having a higher BMI. Previous studies have shown an overall increase in plasma oxylipins in patients with higher BMIs. In the context of the current study, the increase in total plasma oxylipins might have been underrepresented in the STEMI group [52].

We also investigated the oxylipin profile in STEMI patients during different reperfusion time points. As shown in Figure 6, reperfusion resulted in a significant reduction in plasma oxylipins by 24 h, which remained stable for 30 days. However, examination of individual compounds revealed that 17-Keto DHA, 17-Keto DPA, and 20-COOH-AA were three oxylipins that remained elevated post reperfusion. 17-Keto DHA is produced from 17-HDoHE (a docosahexaenoic acid (DHA) metabolite) by a dehydrogenase enzyme, namely 15-hydroxyprostaglandin dehydrogenase (15-PGDH) [53]. 17-Keto DPA is also generated through the same pathways but from docosapentaenoic acid (DPA) [53]. Therefore, the elevated levels of 17-Keto DHA and 17-Keto DPA may reflect an elevated activity of the dehydrogenase enzyme for 30 days post PPCI and/or could reflect a downregulation of their degradation. Both 17-Keto DHA and 17-Keto DPA are PPAR-γ agonists [41], and the activation of Peroxisome Proliferator-Activated Receptor (PPAR)-γ leads to a cascade of events, such as activating Nrf2-dependent antioxidant responses and anti-inflammatory effects by modulating cytokine levels [54]. 20-COOH-AA is also produced by dehydrogenases and is a dual activator of Peroxisome Proliferator-Activated Receptor (PPAR)-α and γ [32] and has vasodilatory effects [51].

The total levels of the metabolites produced through the cytochrome P450 epoxygenase (CYP-e) pathway also did not differ significantly after reperfusion. However, there was a significant reduction in the ratio of n-6-derived epoxides/diols following reperfusion, suggesting that the soluble epoxide hydrolysis (sEH) enzyme activity remained elevated during reperfusion. Interestingly, n-3-derived epoxides/diols did not change during reperfusion. Previous experimental studies showed that increased ratios of EpOME/DiHOME (linoleic acid metabolites) and EpETrEs/DiHETrEs (arachidonic acid metabolites) were associated with a better coronary reactive hyperemic response [55] and vasodilation in coronary and aortas [56]. Also, DiHOMEs were reported to have deleterious effects, including mediating cytotoxic and cardio-depressive effects and causing vascular contraction [57,58]. Furthermore, it was reported that polymorphisms in the soluble epoxide hydrolysis (sEH) gene that lead to higher soluble epoxide hydrolysis (sEH) activity were associated with cardiovascular diseases [59,60]. In contrast, n-3-derived epoxides have been reported to modulate I/R injury. Samokhvalov et al. (2019) [61] demonstrated that the administration of 19,20 EpDPE to HL-1 cardiac cells (cardiac muscle cell line) increased cell viability, elevated mitochondria activity, and improved contractility during hypoxia/reoxygenation. Moreover, they also showed that perfusion of 19,20 EpDPE 20 min before ischemia decreased I/R injury by preserving mitochondrial function and inhibiting nucleotide-like receptor protein 3 (NLRP3) inflammasome activation [40].

Overall, reperfusion resulted in a significant reduction in plasma oxylipin levels in STEMI patients. But 17-keto DHA, 17-Keto DPA, and 20-COOH-AA were three oxylipins that remained elevated post reperfusion. As described above, these secondary metabolites produced by dehydrogenases are elevated in ischemia [32,54], and their continued persistence suggests that these enzymes may be activated and/or those further degrading these metabolites may be inhibited for 30 days post MI. Since all these three metabolites are Peroxisome Proliferator-Activated Receptor (PPAR) agonists [32,51], they may modulate inflammatory and oxidative responses during I/R.

## 5. Conclusions and Future Directions

This study has successfully demonstrated significant changes in oxylipin profiles in patients with STEMI [62]. Given the lack of available therapies to minimize reperfusion injury, potential treatment options could involve increasing levels of oxylipins derived from docosahexaenoic acid (DHA), such as 17-Keto DHA and 17-Keto DPA, as well as inhibiting the soluble epoxide hydrolysis (sEH) enzyme during ischemia or early after reperfusion. A novel approach that has been tested in clinical studies is the acute administration of n-3 fatty acids through intravenous lipid emulsions. Unlike oral supplementation, intravenous administration allows for the rapid integration of these fatty acids into plasma lipids and cell membranes within 30 to 60 min, potentially providing benefits in acute organ injury scenarios. Previous clinical studies have investigated the effects of intravenous infusion of n-3 fatty acids in intensive care patients [63], prior to cardiac surgery [64] and in healthy participants [65]. Therefore, conducting a clinical trial to evaluate the effects of acute administration of DHA emulsion in STEMI patients could yield valuable insights into their impacts. Furthermore, in our study, we also observed increased levels of 20-COOH, 17-Keto DHA, and 17-Keto DPA during myocardial I/R. To further investigate how I/R affects the activity of dehydrogenase enzymes and the levels of their oxylipin metabolites, a series of in vitro and in vivo studies can be designed. Our data also indicate that the ratio of epoxides to diols during ischemia may serve as a potential marker for predicting the size of an infarct. Specifically, we observed that patients with lower ratios of epoxides to diols at baseline tend to have larger infarct sizes. Previous experimental models of myocardial I/R have shown the beneficial effects of soluble epoxide hydrolysis (sEH) enzyme inhibition in I/R injury [66,67,68]. Therefore, a clinical pilot study can be conducted to assess the administration of soluble epoxide hydrolysis (sEH) inhibitor before PPCI in patients presenting with STEMI.

## Figures and Tables

**Table 1:** Characteristics of study participants.

Characteristics	STEMI Patients (n = 45)	Controls (n = 44)	*p*-Value
Male %	66.60	56.30	0.08
Age, yr (mean ± SEM)	65.20 ± 2.08	60.20 ± 1.49	0.06
Body mass index (BMI) (mean ± SEM)	25.80 ± 1.14	30.2 ± 1.05	0.001 *
Left ventricular ejection fraction (LVEF) %	62.00	-	
Time (min), onset of chest pain to reperfusion (Median (min-max))	150 (52–738)	NA	
Left anterior descending coronary artery (LAD) Infarct (%)	41.60	NA	
Right coronary artery (RCA) Infarct (%)	50.00	NA	
Circumflex Infarct (%)	8.00	NA	
Peak CK (Median (min-max)) (units/L)	1105 (141–10,655)	NA	
Peak TnT (Median (min-max)) (ng/L)	1093 (1–10,000)	NA	
Co-morbidity			
Type 2 diabetes mellitus (%)	20.80	14.50	0.40
Smoker (%)	12.70	18.70	0.30
Hypertension (%)	41.60	48.10	0.50
Dyslipidemia (%)	41.60	25.90	0.08
Lipids			
Triglyceride (TG) (mean ± SEM) (mmol/L)	1.70 ± 1.40	-	
High-density lipoprotein (HDL) (mean ± SEM) (mmol/L)	1.20 ± 0.40	-	
Low-density lipoprotein (LDL) (mean ± SEM) (mmol/L)	2.80 ± 0.90	-	
Total cholesterol (mean ± SEM) (mmol/L)	4.20 ± 1.20	-	
Medications at baseline			
ACEI/ARB (%)	20.80	21.80	0.90
Betablocker (%)	6.20	16.30	0.10
Statin (%)	16.60	14.50	0.70

* Significantly different compared with controls (Student’s *t*-test, *p* < 0.05). Abbreviations—ACEI: angiotensin-converting enzyme inhibitors; ARB: angiotensin II receptor blockers; CK: creatine kinase; TnT: troponin T.

**Table 2:** Metabolites that were significantly correlated with markers of cardiac injury during ischemia in STEMI patients (Isch group).

Metabolite	Fatty Acid Precursor	Pathway	CK (r)	*p* Value	TnT (r)	*p* Value
DHA-derived metabolites (total)	NA	NA	0.33	0.046 *	0.50	1.00 × 10^−3^ **
11-HDoHE	DHA	LOX/non-enz	0.56	0.014 **	0.46	0.004 **
13-HDoHE	DHA	LOX/non-enz	0.45	0.005 **	-	-
17-keto DHA	DHA	LOX	-		0.56	0.0003 **
10-HDoHE	DHA	LOX	0.40	0.015 *	0.34	1.00 × 10^−3^ *
16-HDoHE	DHA	LOX	0.42	0.010 *	-	-
17-HDoHE	DHA	LOX	0.37	0.023 *	-	-
8-HDoHE	DHA	LOX	0.33	0.045 *	-	-
16,17 EpDPE	DHA	CYP-e	0.34	0.040 *	0.68	0.000001 **
19,20 DiHDoPE	DHA	CYP-e	-		0.52	0.001 **
20-HDoHE	DHA	CYP-h	0.37	0.025 *	-	-
17-Keto DPA	DPA	LOX	-		0.84	1.00 × 10^−5^ **
5-HETE	AA	LOX	0.40	0.019 *	-	-
11-HETE	AA	LOX	0.43	0.007 **	-	-
12-HETE	AA	LOX	0.41	0.011 *	-	-

The Pearson correlation test was used for the correlation analysis. ** Correlation is significant at the 0.01 level. * Correlation is significant at the 0.05 level. Abbreviations—AA: arachidonic acid; CK: creatine kinase; CYP-e: cytochrome P450 epoxygenase; CYP-h: cytochrome P450 hydroxylase; DHA: docosahexaenoic acid; DiHDoPE: dihydroxy-docosapentaenoic acid; DPA: docosapentaenoic acid; EpDPE: epoxy-docosapentaenoic acid; HDoHE: hydroxy-docosahexaenoic acid; HETE: hydroxy-eicosatetraenoic acid; LOX: lipoxygenase; NA: not applicable; non-enz: non-enzymatic oxidation; TnT: troponin T.

**Table 3:** Plasma levels of oxylipins in Isch and control groups.

Compounds	Fatty Acid	Pathway	Control (nM)	Isch (nM)
PGD2	AA	COX	0.36 ± 0.09	0.00 ± 0.00 *
PGE2	AA	COX	0.09 ± 0.02	0.00 ± 0.00
11,12 DiHETrE	AA	CYP-e	0.35 ± 0.02	0.32 ± 0.04
14,15 DiHETrE	AA	CYP-e	0.35 ± 0.02	0.39 ± 0.05
5,6 DiHETrE	AA	CYP-e	0.15 ± 0.01	0.20 ± 0.03
8,9 DiHETrE	AA	CYP-e	0.20 ± 0.01	0.19 ± 0.03
16-HETE	AA	CYP-h	0.47 ± 0.03	0.29 ± 0.03 *
18-HETE	AA	CYP-h	0.07 ± 0.01	0.11 ± 0.02
20-COOH- AA	AA	CYP-h	37.23 ± 4.11	60.69 ± 7.59 *
20-HETE	AA	CYP-h	0.81 ± 0.09	0.87 ± 0.14
17-HETE	AA	CYP-h	0.04 ± 0.01	0.00 ± 00 *
12-HETE	AA	LOX	2.72 ± 0.43	0.75 ± 0.30 *
5-HETE	AA	LOX	1.80 ± 0.12	3.95 ± 0.92 *
5-oxoETE	AA	LOX	0.21 ± 0.02	0.10 ± 0.01 *
8-HETE	AA	LOX	0.72 ± 0.06	1.00 ± 0.15
tetranor 12-HETE	AA	LOX	0.99 ± 0.10	0.47 ± 0.07 *
12-oxoETE	AA	LOX	0.81 ± 0.19	0.00 ± 0.00 *
15-oxoETE	AA	LOX	0.18 ± 0.02	0.06 ± 0.00 *
15-HETE	AA	LOX/COX	1.73 ± 0.18	1.61 ± 0.32
11-HETE	AA	LOX/COX/non-enz	0.47 ± 0.05	0.44 ± 0.12
9-HETE	AA	LOX/non-enz	0.40 ± 0.07	0.30 ± 0.20
12,13 diHODE	ALA	CYP-e	0.22 ± 0.04	0.16 ± 0.05
12,13 EpODE	ALA	CYP-e	0.20 ± 0.01	0.07 ± 0.00 *
9-oxoOTrE	ALA	LOX	0.70 ± 0.08	0.28 ± 0.03 *
9-HOTrE	ALA	LOX	4.38 ± 0.39	5.29 ± 0.71
15-HETrE	DGLA	LOX	0.39 ± 0.02	0.31 ± 0.06
8-HETrE	DGLA	LOX	0.17 ± 0.01	0.26 ± 0.06
19,20 EpDPE	DHA	CYP-e	0.00 ± 0.00	0.02 ± 0.00 *
19,20 DiHDPE	DHA	CYP-e	1.17 ± 0.09	1.59 ± 0.17
16,17 DiHDPE	DHA	CYP-e	0.07 ± 0.00	0.04 ± 0.00
20-HDoHE	DHA	CYP-h	0.44 ± 0.07	0.52 ± 0.11
10-HDoHE	DHA	LOX	0.25 ± 0.03	0.18 ± 0.04
14-HDoHE	DHA	LOX	0.98 ± 0.22	0.63 ± 0.12
16-HDoHE	DHA	LOX	0.34 ± 0.04	0.27 ± 0.06
17-HDoHE	DHA	LOX	1.02 ± 0.19	1.17 ± 0.23
4-HDoHE	DHA	LOX	1.61 ± 0.28	2.67 ± 0.45
7-HDoHE	DHA	LOX	0.26 ± 0.06	0.57 ± 0.07 *
8-HDoHE	DHA	LOX	0.38 ± 0.07	0.86 ± 0.16 *
17-Keto DHA	DHA	LOX	0.00 ± 0.00	5.30 ± 0.92 *
11-HDoHE	DHA	LOX/non-enz	0.22 ± 0.05	0.06 ± 0.04 *
13-HDoHE	DHA	LOX/non-enz	0.00 ± 0.00	0.24 ± 0.10
17-keto DPA	DPA	LOX	5.21 ± 0.64	4.55 ± 0.82
14,15 diHETE	EPA	CYP-e	1.02 ± 0.10	1.12 ± 0.12
17,18 diHETE	EPA	CYP-e	4.44 ± 0.94	2.67 ± 0.26
18-HEPE	EPA	CYP-h	0.48 ± 0.06	0.56 ± 0.08
12-HEPE	EPA	LOX	0.9 ± 0.23	0.8 ± 0.23
5-HEPE	EPA	LOX	0.49 ± 0.09	1.18 ± 0.17 *
15-HEPE	EPA	LOX/COX	0.29 ± 0.04	0.25 ± 0.04
13-HOTrE	GLA	LOX	3.59 ± 0.40	3.02 ± 0.39
13-HOTrE-y	GLA	LOX	0.65 ± 0.06	0.47 ± 0.06
12,13 diHOME	LA	CYP-e	2.85 ± 0.16	5.99 ± 2.38
12,13 EpOME	LA	CYP-e	3.21 ± 0.22	1.84 ± 0.16 *
9,10 diHOME	LA	CYP-e	1.89 ± 0.12	3.59 ± 0.84
9,10 EpOME	LA	CYP-e	1.22 ± 0.11	0.78 ± 0.10 *
13-HODE	LA	LOX	23.16 ± 1.46	27.48 ± 3.60
13-oxoODE	LA	LOX	5.88 ± 0.45	3.58 ± 0.27 *
9-HODE	LA	LOX	27.53 ± 1.77	31.68 ± 3.61
9-oxoODE	LA	LOX	7.75 ± 0.65	4.05 ± 0.32 *
9,12,13 triHOME	LA	LOX/CYP-e	3.42 ± 0.25	3.58 ± 0.37
9,10,13 triHOME	LA	LOX/CYP-e	9.44 ± 0.70	11.83 ± 0.98

Values are means ± SEM. * Significantly different compared to control group (Student’s *t*-test, *p* < 0.05). (Control n = 44, STEMI n = 45). Abbreviations—AA: arachidonic acid; ALA: alpha-linoleic acid; COX: cyclooxygenase; CYP-e: cytochrome P450 epoxygenase; CYP-h: cytochrome P450 hydroxylase; DGLA: dihomo gamma-linoleic acid; DHA: docosahexaenoic acid; DiHDPE: dihydroxy-docosapentaenoic acid; DiHETE: dihydroxy-eicosatetraenoic acid; DiHETrE: dihydroxy-eicosatrienoic acid; DPA: docosapentaenoic acid; EPA: eicosapentaenoic acid; EpDPE: epoxy-docosapentaenoic acid; EpODE: epoxy-octadecadienoic acid; EpOME: epoxy-octadecenoic acid; GLA: gamma-linoleic acid; HDoHE: hydroxy-docosahexaenoic acid; HEPE: hydroxy-eicosapentaenoic acid; HETE: hydroxy-eicosatetraenoic acid; HETrE: hydroxy-eicosatrienoic acid; HODE: hydroxy-octadecadienoic acid; HOTrE: hydroxy-octadecatrienoic acid; LA: linoleic acid; LOX: lipoxygenase; non-enz: non-enzymatic oxidation; Oxo-ETE: oxo-eicosatetraenoic acid; OxoODE: oxo-octadecadienoic acid; OxoOTrE: oxo-octadecatrienoic acid; PG: prostaglandin; TriHOME: trihydroxy-octadecenoic acid.

## Data Availability

Data is contained within the article or Supplementary Material.

## Supplementary Materials

The following supporting information can be downloaded at: https://www.mdpi.com/article/10.3390/metabo14010019/s1, Table S1: List of oxylipin compounds that were quantified in plasma of by reversed-phase HPLC-MS/MS; Table S2. All quantified oxylipins in STEMI groups during I/R.
