# Supplementary material for: Impact of Reperfusion on Plasma Oxylipins in ST-Segment Elevation Myocardial Infarction
Source: Metabolites. 2023 Dec 27;14(1):19. doi: 10.3390/metabo14010019 (PMC10821107; doi:10.3390/metabo14010019)
Supplement: Supplementary file 1 [file metabolites-14-00019-s001.zip › supplementary data.pdf]

Impact of reperfusion on plasma oxylipins in ST-segment Elevation Myocardial Infarction

Zahra Solati[1,2], Arun Surendran[1,2], Harold M Aukema[1,3,4], and Amir Ravandi[1,2,5]

[1]Precision Cardiovascular Medicine Group; St. Boniface Hospital, Albrechtsen Research Centre,
[2]Department of Physiology and Pathophysiology, Rady Faculty of Health Sciences, University of Manitoba
[3]Department of Food and Human Nutritional Sciences, University of Manitoba
[4]Canadian Centre for Agri-Food Research in Health and Medicine, St. Boniface Hospital Research Centre, Winnipeg, Canada.
[5] Section of Cardiology, University of Manitoba, Winnipeg, Canada.

Corresponding author & Lead Contact
Dr. Amir Ravandi MD PhD FRCPC
Interventional Cardiology
Cardiovascular Lipidomics Laboratory,
St. Boniface Hospital,
409 Tache Ave,
Winnipeg, MB Canada R2H 2A6

Phone.204-235-3206 and 204-235-3414
Fax.204-235-0793 and 204-235-0793

Email: aravandi@sbgh.mb.ca

Table S1: List of oxylipin compounds that were quantified in plasma of by reversed-phase HPLC-MS/MS

Analyte	Pathway	Fatty Acid	Q1 Mass (Da)	Q3 Mass (da)	Internal standard name	Retention time (min)	Response factor	LLOD in 100ul (ng/ul)	ULOD in 100ul (ng/ul)
**PGD2**	COX	AA	351	271	(d4) PGD2.IS	7.91	2.28045	0.003	22.22
**PGE2**	COX	AA	351	271	(d4) PGE2.IS	7.59	8.68273	0.003	22.22
**5-HETE**	LOX	AA	319	115	(d8) 5-HETE.IS	17.13	0.90038	0.0002	22.22
**5-oxoETE**	LOX	AA	317	203	(d7) 5-oxoETE.IS	17.5	1.04473	0.0002	22.22
**8-HETE**	LOX	AA	319	155	(d8) 5-HETE.IS	16.66	1.24638	0.0009	22.22
**9-HETE**	LOX	AA	319	123	(d8) 5-HETE.IS	16.8	0.29958	0.003	22.22
**11-HETE**	LOX	AA	319	167	(d8) 12-HETE.IS	16.39	7.67408	0.0009	22.22
**12-HETE**	LOX	AA	319	135	(d8) 12-HETE.IS	16.56	0.15621	0.003	22.22
**12-oxoETE**	LOX	AA	317	153	(d7) 5-oxoETE.IS	16.54	0.12689	0.04	22.22
**tetranor-12-HETE**	LOX	AA	265	109	(d8) 12-HETE.IS	13.29	2.40204	0.0002	22.22
**15-HETE**	LOX	AA	319	175	(d8) 15-HETE.IS	16.05	0.85626	0.003	22.22
**15-oxoETE**	LOX	AA	317	113	(d7) 5-oxoETE.IS	16.11	3.26434	0.0002	22.22
**9-HOTrE**	LOX	ALA	293	171	(d4) 9-HODE.IS	14.46	1.50163	0.003	22.22
**9-oxoOTrE**	LOX	ALA	291	185	(d7) 5-oxoETE.IS	14.96	2.2951	0.0002	22.22
**13-HOTrE**	LOX	ALA	293	195	(d4) 13-HODE.IS	14.6	1.15092	0.003	22.22
**5-HEPE**	LOX	EPA	317	115	(d8) 5-HETE.IS	15.79	0.68513	0.003	22.22
**12-HEPE**	LOX	EPA	317	179	(d8) 12-HETE.IS	15.32	1.73582	0.003	22.22
**15-HEPE**	LOX	EPA	317	219	(d8) 15-HETE.IS	14.98	1.56695	0.148	22.22
**4-HDoHE**	LOX	DHA	343	101	(d8) 5-HETE.IS	17.32	0.39766	0.003	22.22
**7-HDoHE**	LOX	DHA	343	141	(d8) 5-HETE.IS	16.66	0.51513	0.0009	22.22
**8-HDoHE**	LOX	DHA	343	109	(d8) 5-HETE.IS	16.73	0.25003	0.003	22.22
**10-HDoHE**	LOX	DHA	343	153	(d8) 12-HETE.IS	16.35	1.68431	0.0002	22.22
**14-HDoHE**	LOX	DHA	343	205	(d8) 15-HETE.IS	16.29	0.78401	0.003	22.22
**16-HDoHE**	LOX	DHA	343	233	(d8) 15-HETE.IS	15.97	3.11823	0.0009	22.22
**17-HDoHE**	LOX	DHA	343	245	(d8) 15-HETE.IS	16.03	0.2437	0.042	22.22
**17keto-DHA**	LOX	DHA	341	297	(d7) 5-oxoETE.IS	16.28	0.11372	0.042	22.22
**11-HDoHE**	LOX/non-enz	DHA	343	149	(d8) 12-HETE.IS	16.48	0.88676	0.003	22.22
**13-HDoHE**	LOX/non-enz	DHA	343	221	(d8) 12-HETE.IS	16.17	0.50603	0.003	22.22
**17-Keto DPA**	LOX	DPA	343	247	(d7) 5-oxoETE.IS	16.86	0.1154	0.012	22.22
**9-HODE**	LOX	LA	295	171	(d4) 9-HODE.IS	15.93	1.33961	0.0002	22.22
**9-oxoODE**	LOX	LA	293	185	(d7) 5-oxoETE.IS	16.26	0.95769	0.012	22.22
**13-HODE**	LOX	LA	295	195	(d4) 13-HODE.IS	15.78	3.34877	0.0002	22.22
**13-oxoODE**	LOX	LA	293	167	(d7) 5-oxoETE.IS	15.95	0.65942	0.012	22.22

**9,10,13-triHOME**	LOX/CYP-e	LA	329	171	(d4) 9,10 diHOME.IS	7.44	39.48725	0.0002	22.22
**9,12,13-triHOME**	LOX/CYP-e	LA	329	211	(d4) 12,13 diHOME.IS	7.38	61.41712	0.0002	22.22
**13-HOTrE-γ**	LOX	GLA	293	193	(d4) 13-HODE.IS	14.88	3.26494	0.0034	22.22
**8-HETrE**	LOX	DGLA	321	157	(d8) 5-HETE.IS	17.07	1.87458	0.0009	22.22
**15-HETrE**	LOX	DGLA	321	221	(d8) 15-HETE.IS	16.7	3.79631	0.0009	22.22
**5,6-DiHETrE**	CYP-e	AA	337	145	(d11) 8,9 DiHETrE.IS	15.55	1.78532	0.0034	22.22
**8,9-DiHETrE**	CYP-e	AA	337	127	(d11) 8,9 DiHETrE.IS	14.85	1.77545	0.0034	22.22
**11,12-DiHETrE**	CYP-e	AA	337	167	(d11) 11,12 DiHETrE.IS	14.27	9.12136	0.0034	22.22
**14,15-DiHETrE**	CYP-e	AA	337	207	(d11) 14,15 DiHETrE.IS	13.56	8.93326	0.00028	22.22
**12,13-EpODE**	CYP-e	ALA	293	183	(d4) 12,13 diHOME.IS	16.17	21.70636	0.0009	22.22
**12,13-diHODE**	CYP-e	ALA	311	183	(d4) 12,13 diHOME.IS	11.24	34.86462	0.00028	22.22
**16,17-DiHoDPE**	CYP-e	DHA	361	233	(d11) 14,15 DiHETrE.IS	13.93	6.23301	0.00098	22.22
**19,20-EpDoPE**	CYP-e	DHA	343	241	(d11) 14,15 DiHETrE.IS	16.87	1.10022	0.012	22.22
**19,20-DiHDoPE**	CYP-e	DHA	361	229	(d11) 14,15 DiHETrE.IS	13.35	0.91613	0.012	22.22
**9,10-EpOME**	CYP-e	LA	295	171	(d4) 9,10 diHOME.IS	17.35	37.33091	0.0009	22.22
**9,10-diHOME**	CYP-e	LA	313	201	(d4) 9,10 diHOME.IS	13.24	79.72576	0.0009	22.22
**12,13-EpOME**	CYP-e	LA	295	195	(d4) 12,13 diHOME.IS	17.17	22.85861	0.0034	22.22
**12,13-diHOME**	CYP-e	LA	313	183	(d4) 12,13 diHOME.IS	12.71	34.6768	0.00028	22.22
**16-HETE**	CYP-h	AA	319	189	(d8) 15-HETE.IS	15.5	0.72228	0.0009	22.22
**17-HETE**	CYP-h	AA	319	247	(d8) 15-HETE.IS	15.4	2.73902	0.0034	22.22
**18-HETE**	CYP-h	AA	319	261	(d6) 20-HETE.IS	15.26	2.58404	0.0034	22.22
**19-HETE**	CYP-h	AA	319	231	(d6) 20-HETE.IS	14.88	0.17759	0.148	22.22
**20-HETE**	CYP-h	AA	319	245	(d6) 20-HETE.IS	15.01	0.32666	0.0034	22.22
**20-COOH-AA**	CYP-h	AA	333	271	(d5) EPA.IS	14.41	0.64699	0.012	22.22
**18-HEPE**	CYP-h	EPA	317	215	(d8) 15-HETE.IS	14.49	1.1901	0.003	22.22
**20-HDoHE**	CYP-h	DHA	343	241	(d6) 20-HETE.IS	15.7	0.8816	0.012	22.22

Abbreviations: AA: Arachidonic acid, ALA: Alpha linoleic acid, COX: Cyclooxygenase, CYP-e: Cytochrome P450 epoxygenase, CYP-h: Cytochrome P450 hydroxylase, DGLA: Dihemo gamma-linoleic acid, DHA: Docosahexaenoic acid, DiHDPE: Dihydroxydocosapentaenoic acid, DiHETE: Dihydroxyeicosatetraenoic acid, DiHETrE: Dihydroxy-eicosatrienoic acid, DPA: Docosapentaenoic acid, EPA: Eicosapentaenoic acid, EpDPE: Epoxydocosapentaenoic acid, EpODE: Epoxy-octadecadienoic acid, EpOME: Epoxy-octadecenoic acid, GLA: Gamma-linolenic acid, HDoHE: Hydroxy-docosahexaenoic acid, HEPE: Hydroxyeicosapentaenoic acid, HETE: Hydroxyeicosatetraenoic acid, HETrE: Hydroxyeicosatrienoic acid, HODE: Hydroxyoctadecadienoic acid, HOTrE: Hydroxy-octadecatrienoic acid, LA: Linoleic acid, LLOD: Lower limit of detection, LOX: Lipoxygenase, MPO: Myeloperoxidase, non-enz: Non-enzymatic oxidation, Oxo-ETE: Oxo-eicosatetraenoic acid, OxoODE: Oxo-octadecadienoic acid, OxoOTrE: Oxo-octadecatrienoic acid, PG: Prostaglandin, TriHOME: Trihydroxy-octadecenoic acid,ULOD: Upper limit of detection.

Table S2. All quantified oxylipins in STEMI groups during I/R.

Compounds	FA	Pathway	Isch (nM)	R-2h(nM)	R-24h(nM)	R-48h(nM)	R-30d(nM)
11,12 DiHETrE	AA	CYP-e	0.32±0.04	0.35±0.05	0.23±0.03	0.25±0.03	0.33±0.02
14,15 DiHETrE	AA	CYP-e	0.39±0.05	0.40±0.05	0.24±0.03	0.23±0.02 b	0.33±0.02
5,6 DiHETrE	AA	CYP-e	0.20±0.03	0.19±0.03	0.08±0.01 a,b	0.08±0.01 a,b	0.11±0.01
8,9 DiHETrE	AA	CYP-e	0.19±0.03	0.20±0.04	0.10±0.01	0.09±0.01 b	0.13±0.01
16-HETE	AA	CYP-h	0.29±0.03	0.27±0.03	0.20±0.02	0.20±0.02	0.33±0.03
18-HETE	AA	CYP-h	0.11±0.02	0.15±0.03	0.04±0.01 b	0.03±0.01 b	0.05±0.01 b
20-COOH-AA	AA	CYP-h	60.69±7.59	73.59±11.27	46.92±6.28	50.57±5.86	65.58±11.03
20-HETE	AA	CYP-h	0.87±0.14	0.69±0.09	0.39±0.05 a	0.38±0.05 a	0.54±0.13
12-HETE	AA	LOX	0.75±0.30	0.52±0.16	0.46±0.14	0.53±0.16	0.12±0.07 a
5-HETE	AA	LOX	3.95±0.92	2.51±0.25	1.33±0.19 a	1.44±0.18 a	1.21±0.21 a
5-oxoETE	AA	LOX	0.10±0.01	0.07±0.01	0.03±0.007 a	0.03±0.01 a	0.05±0.01
8-HETE	AA	LOX	1.00±0.15	0.70±0.07	0.57±0.06 a	0.54±0.06 a	0.62±0.14
tetranor 12-HETE	AA	LOX	0.47±0.07	0.65±0.10	0.38±0.05 b	0.29±0.04 b	0.38±0.04
15-HETE	AA	LOX/COX	1.61±0.32	1.15±0.14	0.8±0.11 a	0.8±0.09 a	0.58±0.07 a
11-HETE	AA	LOX/COX/non-enzyme	0.44±0.12	0.25±0.05	0.16±0.04 a	0.15±0.04 a	0.13±0.02 a
9-HETE	AA	LOX/non-enz	0.30±0.20	0.10±0.06	0.10±0.05	0.12±0.06	0.27±0.13
15-oxoETE	AA	LOX	0.06±0.01	0.05±0.01	0.02±0.00 a	0.03±0.00 a	0.02±0.01 a
12,13 diHODE	ALA	CYP-e	0.16±0.05	0.1±0.01	0.09±0.01	0.13±0.03	0.17±0.02
12,13 EpODE	ALA	CYP-e	0.07±0.01	0.09±0.01	0.06±0.01	0.08±0.03	0.07±0.01
9 oxoOTrE	ALA	LOX	0.28±0.03	0.28±0.04	0.10±0.02 a,b	0.10±0.03 a,b	0.09±0.02 a,b
9-HOTrE	ALA	LOX	5.29±0.71	4.59±0.71	1.84±0.22 a,b	1.84±0.29 a,b	2.32±0.34 a,b
15-HETrE	DGLA	LOX	0.31±0.06	0.21±0.03	0.09±0.01 a	0.12±0.02 a	0.10±0.01 a
8-HETrE	DGLA	LOX	0.26±0.06	0.17±0.02	0.07±0.01 a	0.08±0.01 a	0.09±0.01 a
16,17 DiHDPE	DHA	CYP-e	0.04±0.01	0.04±0.01	0.03±0.00	0.03±0.00	0.04±0.01
16,17 EpDPE	DHA	CYP-e	0.03±0.02	0.03±0.02	0.01±0.01	0.01±0.01	0.06±0.06
19,20 DiHDPE	DHA	CYP-e	1.59±0.17	1.72±0.26	0.93±0.12 a,b	0.89±0.11 a,b	0.83±0.07 a,b
19,20 EpDPE	DHA	CYP-e	0.02±0.01	0.01±0.01	0.001±0.00 a	0.002±0.00 a	0.008±0.01
20-HDoHE	DHA	CYP-h	0.52±0.11	0.35±0.06	0.21±0.05 a	0.17±0.05 a	0.14±0.05 a
10-HDoHE	DHA	LOX	0.18±0.04	0.08±0.01	0.08±0.03	0.05±0.01 a	0.08±0.02
14-HDoHE	DHA	LOX	0.63±0.12	0.35±0.05	0.28±0.05 a	0.26±0.05 a	0.22±0.04 a
16-HDoHE	DHA	LOX	0.27±0.06	0.16±0.03	0.12±0.03	0.09±0.02 a	0.09±0.02 a
17-HDoHE	DHA	LOX	1.17±0.23	0.84±0.14	0.58±0.10 a	0.53±0.09 a	0.27±0.15 a
17-keto DHA	DHA	LOX	5.30±0.92	4.27±0.63	6.74±1.25	6.36±1.09	3.87±0.70
4-HDoHE	DHA	LOX	2.67±0.45	1.68±0.23	0.82±0.09 a	0.96±0.09 a	0.80±0.16 a
7-HDoHE	DHA	LOX	0.57±0.07	0.37±0.06	0.25±0.04 a	0.26±0.05 a	0.20±0.08 a
8-HDoHE	DHA	LOX	0.86±0.16	0.69±0.11	0.30±0.09 a	0.28±0.07 a	0.26±0.11 a
11-HDoHE	DHA	LOX/non-enz	0.06±0.04	0±0.00	0.03±0.03	0.00±0.00	0.00±0.00
13-HDoHE	DHA	LOX/non-enz	0.24±0.10	0.06±0.03	0.08±0.04	0.02±0.02	0.00±0.00
17-keto DPA	DPA	LOX	4.55±0.82	4.08±0.49	4.08±0.99	3.97±0.77	2.89±0.85
14,15 diHETE	EPA	CYP-e	1.12±0.12	1.12±0.21	0.77±0.09	0.63±0.05	0.65±0.04
17,18 diHETE	EPA	CYP-e	2.67±0.26	2.63±0.39	1.6±0.19 a,b	1.57±0.18 a,b	1.18±0.10 a,b
18-HEPE	EPA	CYP-h	0.56±0.08	0.41±0.06	0.15±0.03 a,b	0.09±0.01 a,b	0.14±0.02 a,b

12-HEPE	EPA	LOX	0.80±0.23	0.47±0.07	0.15±0.03 a	0.10±0.03 a	0.08±0.02 a
5-HEPE	EPA	LOX	1.18±0.17	0.94±0.14	0.38±0.07 a,b	0.18±0.05 a,b	0.29±0.08 a,b
15-HEPE	EPA	LOX/COX	0.25±0.04	0.19±0.04	0.07±0.01 a	0.07±0.01a	0.08±0.01 a
13-HOTrE	GLA	LOX	3.02±0.39	2.35±0.28	1.84±0.32 a	1.50±0.18 a	2.07±0.24
13-HOTrE-y	GLA	LOX	0.47±0.06	0.34±0.05	0.11±0.02 a,b	0.10±0.01 a,b	0.19±0.03 a
9,10 diHOME	LA	CYP-e	3.59±0.84	3.05±0.49	2.86±0.91	3.93±1.86	3.21±0.40
12,13 EpOME	LA	CYP-e	1.84±0.16	2.16±0.20	1.32±0.16 b	1.38±0.25 b	1.12±0.13 b
12,13 diHOME	LA	CYP-e	5.99±2.38	3.96±0.42	3.78±0.55	4.25±1.24	4.97±0.60
9,10 EpOME	LA	CYP-e	0.78±0.10	0.83±0.10	0.39±0.07	0.51±0.16	0.34±0.08 b
13-HODE	LA	LOX	27.48±3.60	23.23±2.84	14.82±2.03 a	14.19±1.98 a	13.63±1.19 a
13-oxoODE	LA	LOX	3.58±0.27	3.09±0.27	2.06±0.18 a,b	2.15±0.25 a,b	1.77±0.16 a,b
9-HODE	LA	LOX	31.68±3.61	28.23±3.75	13.18±1.62 a,b	13.41±1.97 a,b	12.59±1.4 a,b
9-oxoODE	LA	LOX	4.05±0.32	4.12±0.42	1.87±0.24 a,b	2.05±0.27 a,b	1.5±0.20 a,b
9,10,13 triHOME	LA	LOX/CYP-e	11.83±0.98	9.01±0.85	6.83±0.67 a	7.00±0.61 a	5.87±0.70 a
9,12,13 triHOME	LA	LOX/CYP-e	3.58±0.37	2.74±0.24	2.43±0.27	2.31±0.22 a	2.59±0.31

Values are means ± SEM. a Significant difference from Isch, b Significant difference from R-2h (One way analysis of variance (ANOVA) with a post-hoc Tukey test for multiple comparisons was used to determine statistical significance between study groups, ($p<0.05$). (Isch, n=45), (R-2h, n=42), (R-24h, n=442), (R-48h, n=43), (R-30d, n=29), Control n=44). Abbreviations: AA: Arachidonic acid, ALA: Alpha-linoleic acid, COX: Cyclooxygenase, CYP-e: Cytochrome P450 epoxygenase, CYP-h: Cytochrome P450 hydroxylase, DGLA: Dihomo gamma-linoleic acid, DHA: Docosahexaenoic acid, DiHDPE: Dihydroxy-docosapentaenoic acid, DiHETE: Dihydroxy-eicosatetraenoic acid, DiHETrE: Dihydroxy-eicosatrienoic acid, DPA: Docosapentaenoic acid, EPA: Eicosapentaenoic acid, EpDPE: Epoxy-docosapentaenoic acid, EpODE: Epoxy-octadecadienoic acid, EpOME: Epoxy-octadecenoic acid, GLA: Gamma-linoleic acid, HDoHE: Hydroxy-docosahexaenoic acid, HEPE: Hydroxy-eicosapentaenoic acid, HETE: Hydroxy-eicosatetraenoic acid, HETrE: Hydroxy-eicosatrienoic acid, HODE: Hydroxy-octadecadienoic acid, HOTrE: Hydroxy-octadecatrienoic acid, LA: Linoleic acid, LOX: Lipoxygenase, non-enz: Non-enzymatic oxidation, Oxo-ETE: Oxo-eicosatetraenoic acid, OxoODE: Oxo-octadecadienoic acid, OxoOTrE: Oxo-octadecatrienoic acid, PG: Prostaglandin, TriHOME: Trihydroxy-octadecenoic acid.

Separation Workflow

Created by https://lipidomicstandards.org, version v2.3.2

Overall study design

Title of the study	Impact of reperfusion on plasma oxylipins in ST-segment Ele-vation Myocardial Infarction		
Document creation date	11/20/2023	Corresponding Email	amir.ravandi@umanitoba.ca
Principle investigator	Amir Ravandi	Is the workflow targeted or untargeted?	Targeted
Institution	Precision Cardiovascular Medicine Group; St. Boniface Hospital, Albrechtsen Research Centre, 2Department of Physiology and Pathophysiology, Rady Faculty of Health Sciences, University of Manitoba	Clinical	Yes

Lipid extraction

Extraction method	Solid-Phase Extraction	Solid-Phase Extraction	Reverse-Phase
pH adjustment	Hydrochloric acid	Were internal standards added prior extraction?	Yes

Analytical platform

Which solvents were used	water/acetonitrile/acetic acid	Ion source	ESI
Number of separation dimensions	One dimension	MS Level	MS2
Separation type 1	LC	Mass window for precursor ion isolation (in Da total isolation window)	1
Separation mode 1 (liquid)	RP	Mass resolution for detected ion at MS2	Low resolution
Detector	Mass spectrometer	Resolution at MS2	Low
MS type	QQQ	Was/Were additional dimension/techniques used	No
MS vendor	SCIEX		

Quality control

Blanks	Yes	Quality control	Yes
Type of Blanks	Extraction blank, Injection blank	Type of QC sample	Sample pool

Method qualification and validation

Method validation	Yes	Precison	No
Lipid recovery	No	Accuracy	Yes
Dynamic quantification range	Yes	Guidelines followed	None
Limit of quantitation (LOQ)/Limit of detection (LOD)	Yes		

Reporting

Are reported raw data uploaded into repository?	Available on request	Raw data upload	Available on request
Are metadata available?	No	Additional comments	-

Sample Descriptions

Plasma Oxylipins / Human / Plasma

Provided information	-	Additives	EDTA
Temperature handling original sample	4-8 °C	Were samples stored under inert gas?	No
Instant sample preparation	Yes	Additional preservation methods	No
Storage temperature	-80 °C	Biobank samples	No
